# Acute Myocardial Infarction and Daylight Saving Time Transitions: Is There a Risk?

**DOI:** 10.3390/clockssleep3040039

**Published:** 2021-10-25

**Authors:** Viktor Čulić, Thomas Kantermann

**Affiliations:** 1 Department of Cardiology and Angiology, University Hospital Center Split, 21000 Split, Croatia; 2 Department of Clinical Propaedeutics, University of Split School of Medicine, 21000 Split, Croatia; 3 Faculty for Health and Social Affairs, University of Applied Sciences for Economics and Management (FOM), 45127 Essen, Germany; thomas.kantermann@fom.de; 4 SynOpus, 44799 Bochum, Germany

**Keywords:** acute myocardial infarction, chronotypes, circadian misalignment, daylight saving time, sex, sleep deprivation

## Abstract

Available evidence on the risk of acute myocardial infarction (AMI) in the days after the spring daylight saving time (DST) transition suggests either a modest increase or no risk increase. Partial sleep deprivation and enhanced circadian clock misalignment have been implicated as the underlying mechanisms for increased AMI risk, probably via enhanced thrombo-inflammatory processes and activation of the sympathetic nervous system. Most of the studies, as we suggest as a perspective here, have used potentially inappropriate control periods, including the two post-transitional weeks, because adjustment after the spring DST transition lasts at least four weeks for all chronotypes and probably even beyond this period for late chronotypes. The most plausible conclusions, at the moment, for the risk of AMI after the spring DST transition are: (1) the risk is increased, (2) a relatively modest risk increase could be currently underestimated or in some studies undetected, (3) late chronotypes and/or individuals with high levels of social jetlag (a proxy for circadian clock misalignment) could be more affected by the phenomenon, and (4) underlying pathophysiological mechanisms should be further explored. As a significant part of world’s population continues to be affected by the biannual clock change, the question of increased AMI risk in the post-transitional period remains an intriguing public health issue.

## 1. Significance of the Phenomenon and Unanswered Questions—A Perspective

Our brainstem nuclei, the hypothalamic–pituitary–adrenal axis, and autonomic nervous system, through the cardiovascular, immune, and metabolic systems, regulate the physiological adaptation to everyday circumstances and challenges. In coronary patients, daily life events, psycho-emotional, and environmental factors may induce a maladaptive response, causing a disruption of vulnerable coronary plaques and leading to acute myocardial infarction (AMI) [1,2]. Since the first report in 2008 [3], studies have suggested an increase in number of AMIs in the days after the spring daylight saving time (DST) transition [4,5,6]. Although not all studies have reported an overall significant effect [7,8,9] (Table 1), a 2019 meta-analysis showed a 3% risk increase in the post-transitional week [10].

DST is currently applied in over 70 countries worldwide, impacting more than 1.5 billion people or a quarter of the world’s population, so any impact of DST on human health is potentially highly significant even if the effect size is small. Considering the risk of AMI after the DST spring transition, there are several principal questions that require answers: (1) does it exist; (2) if it does, to what extent does it affect the general population; (3) does it affect all individuals equally; and (4) what are the underlying mechanisms? As discussed below, there is a strong possibility that previous studies from this field did not use appropriate control periods and average the effect across chronotypes, thereby blunting and underestimating the measured effect and producing inconsistent findings.

## 2. Circadian Clock, Chronotypes and DST

The circadian clock synchronizes physiological and behavioral rhythms to the environmental light/dark cycle. The relationship between environmental and endogenous (biological) time is called the phase of entrainment [11]. Depending on age, sex, genetic makeup, geographical location of residence, and light exposure, people differ in their phase of entrainment, i.e., have different chronotypes [12,13]. Compared with early chronotypes, late chronotypes may be at greater cardiovascular risk due to their higher susceptibility towards diabetes and hypertension independent of sleep duration or sleep sufficiency [14]. Regardless of the DST transitions, late chronotypes also show, on average, higher levels of social jetlag—a proxy for the misalignment between their biological clocks and the environment [15]. Even in apparently healthy individuals, social jetlag may contribute to cardiovascular risk through its association with physical inactivity, increased cortisol levels, and higher resting heart rate [14,16,17].

Our body clocks systematically adjust to changes in photoperiod, but by suddenly changing the social clocks by one hour we abruptly change the phase angle between the time of arising and dawn [18]. Even without a change in time of arising caused by DST, many individuals experience circadian misalignment coupled with sleep loss [15,19,20], which has been associated with an increase in the risk of cardiovascular diseases [21,22]. Superimposed abrupt circadian misalignment caused by the spring DST transition might therefore pose additional cardiovascular burden on individuals with high levels of social jetlag, in primarily late chronotypes.

## 3. Previous Research: The Problem of Control Period in Spring

An appropriate control group or period in clinical research is essential to demonstrate that a diagnostic method, treatment, intervention, or exposure has a different effect compared to the standard procedures or conditions. Controls should be selected on the basis of comparability to the population at risk and ideally are free from the influence of the diagnostic method, treatment, intervention, or exposure of interest. In terms of exposure, a misclassification of cases inevitably produces statistical noise and bias.

In assessment of the effect of the risk associated with DST, it should be kept in mind that the influence of the transition, i.e., the hazard period, is transient and most likely variable between individuals. An immediate powerful effect may in some individuals be followed by a milder but longer lasting effect that fades out slowly.

**Figure 1:**
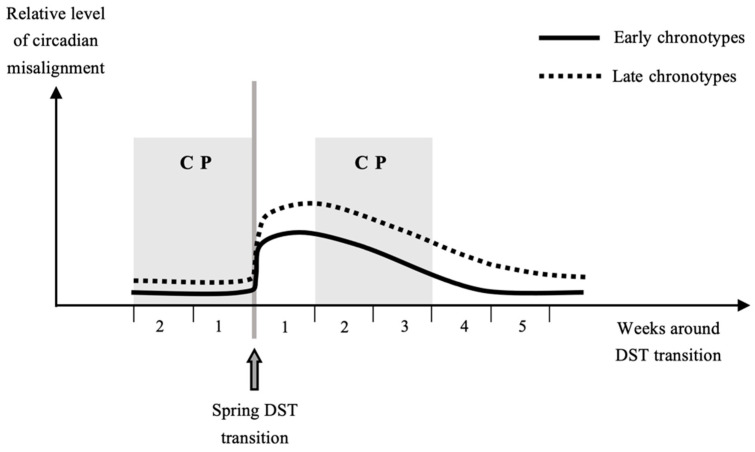
Circadian misalignment in relation to the spring daylight saving time (DST) transition in early and late chronotypes. Late chronotypes at baseline have relatively greater levels of circadian misalignment. Control periods (CPs) are shown as applied in the majority of studies on the post-DST incidence of acute myocardial infarction. Physiological adaptation is expected to be longer, in late chronotypes more than four weeks, and the two post-transitional CP weeks fall within this period. Instead of being used as CP, those weeks could represent the hazard period of an increased cardiovascular risk.

Lasting approximately a week, circadian clock adjustment after the autumn DST transition appears less problematic in all chronotypes [18]. In contrast, adjustment after the spring transition may last at least four weeks, whereas late chronotypes likely continue to adjust their circadian clocks even beyond this period (Figure 1) [18]. The study exploring this issue [18] consisted of two parts, with (i) data mining of a large database containing 55,000 participants from Central Europe and (ii) a longitudinal study (with sleep diaries and actigraphy) to describe the adjustment process to the DST transition at the individual level in 50 participants, studied for four weeks before and four weeks after both the autumn and the spring, respectively. The results showed that the circadian system in humans did not adjust to DST and further, that the usually occurring seasonal adjustment to changing photoperiods was disturbed by the transition to DST [18].

In a recent study from the University of Michigan, sleep patterns after the spring DST have been investigated to assess how individual differences on a genetic level affect an individual’s ability to adjust to such an abrupt shift. The study included medical interns, and polygenic scores were used to differentiate individual genomic predisposition to morningness or eveningness [23]. While those who were genetically predisposed to morningness were able to return to a normal sleep pattern and recover from the hour they lost three days after the transition, those with evening circadian inclination in turn exhibited significantly increased social jet lag and shorter average time asleep, and they had not shifted to the time transition for a whole week after the spring DST [23]. This study strongly supports the chronotype differences in adaptation to spring DST. As the study was limited to the comparison between the pre- and the post-transitional weeks, it did not explore the duration of observed disturbances in evening-inclined individuals.

The most important problem is that the duration of hazard and control periods in most of the studies on the AMI incidence in the days after the DST transitions was not justified and determined empirically by a sensitivity analysis. Instead, the duration of hazard and control periods was chosen following the arbitrarily determined periods that have been used in the first landmark study in this field [3]. Most of the subsequent studies also used two weeks before and two weeks after the post-transitional week as control periods [4,5,6,8,9]. This procedure seems (coincidentally) appropriate for the autumn shift, but the post-DST control period in spring falls into the adjustment period for all chronotypes (Figure 1). By this approach, a number of people who may still undergo the process of adaptation could be incorporated among the controls. Such an inappropriate control period likely leads to an underestimation of the spring DST effect on AMI, particularly in the recent Dutch study observing no risk increase and including only the second post-transitional week as one of the 3 control weeks [7].

## 4. Interactions among Chronotypes, Sex and Geographical Locations

Due to the complexity of the circadian entrainment process, chronotype-specific differences in cardiovascular risk can also be suspected from findings on differences in DST-associated risk of AMI, due to sex and geographical location. For most of their adulthood, men are, on average, later chronotypes than women [24]. This chronotype difference is most pronounced in younger years and disappears with increasing age [24]. Additionally, preliminary results suggest that latitude influences chronotype in a specific manner: adolescents in the tropics are the earliest, those in colder climates are later, and those in the subtropics are the latest sleepers [25].

Studies in southern German [8] and southern Croatian [5] populations suggest that AMI incidence is increased after the autumn DST transition in women [8], whereas both studies [5,8] show that men, in turn, are more vulnerable to the spring DST transition. In one Swedish study women were more affected by the spring DST transition, while men were more affected by the autumn DST transition [3]. Compared with other investigated regions [3,4,6,7,8,9], south Croatia is nearest the subtropics and men from this region showed the greatest vulnerability to the spring DST transition [5]. However, another Swedish [6], Finish [9], and Dutch [7] studies revealed no sex differences in vulnerability to the DST transitions.

## 5. AMI and DST: Possible Underlying Pathophysiological Mechanisms

Accumulating evidence suggests that both increased systemic inflammation and hemodynamic-associated biomechanical forces can compound the chain of events, leading to destabilization of an atherosclerotic coronary plaque already predisposed to provoke an AMI onset [1]. The sympathetic nervous system activation produces biomechanical forces such as increased cardiac contractility, twisting and bending of the coronary arteries, heart rate, blood pressure, circumferential stress, and coronary vasoconstriction—forces often responsible for plaque disruption, and at the same time stimulates platelet activation and hypercoagulability [1,2]. Increased inflammatory processes further increase the thrombogenic potential of the atherosclerotic plaque and facilitate the thrombus formation [1].

Acute cardiovascular effects of the DST transitions have not been specifically explored as of yet. In turn, sleep deprivation and enhanced circadian clock misalignment have been implicated as forerunners of the AMI risk [3,4,5,6,10]. Circadian clock misalignment contributes to a wide range of human diseases, especially in those where the disease-related events are substantially influenced by the time of day, such as AMI [26]. Waking up one hour earlier after the spring DST transition both shortens sleep and could lead to increased arousal at an underprepared physiological time, which in combination could introduce the tipping point, leading to an increased AMI risk.

**Figure 2:**
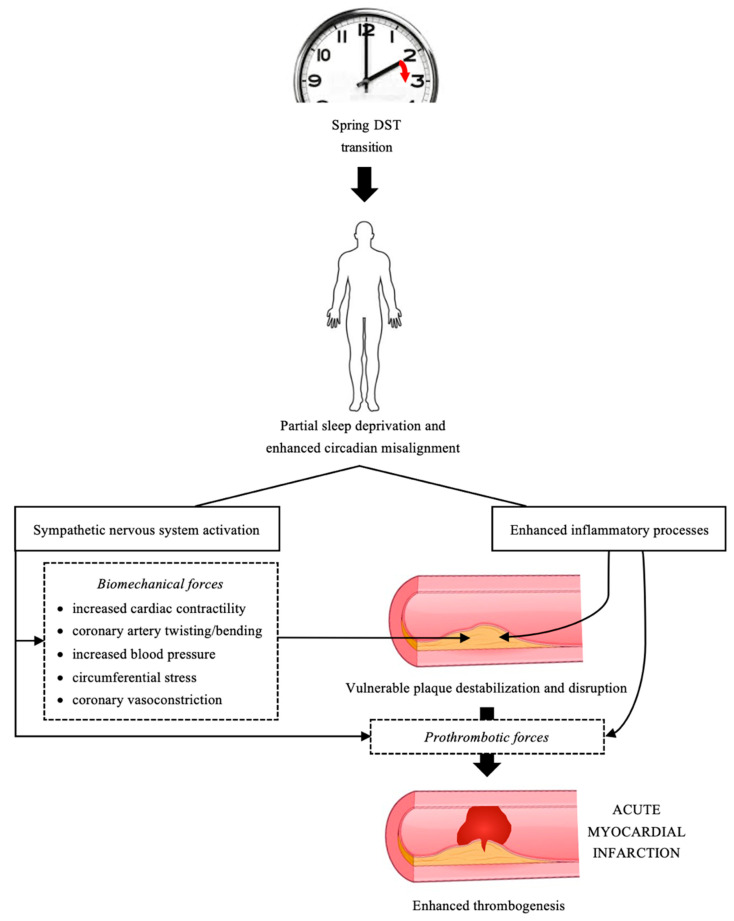
Mechanisms of increased risk of acute myocardial infarction in the period after the spring daylight saving time (DST) transition. Increased inflammation coupled with enhanced hemodynamic and biomechanical forces induced by sympathetic nervous system arousal can cause destabilization of vulnerable coronary atherosclerotic plaque. As those processes accompany the circadian clock misalignment and partial lack of sleep and last over a several week period, they could initiate a superficial erosion of the intimal surface or rupture of a vulnerable plaque. Increased inflammation and sympathetic nervous system activity can also initiate formation of an overlaying thrombus and/or facilitate enhanced thrombus growth, to produce a more significant coronary occlusion during the infarction onset.

Studies on short-term acute partial sleep deprivation provide an insight into possible mechanisms of increased AMI risk (Figure 2). It is to be noted that these mechanisms are not very intense in their effect, but over the course of post-transitional days could destabilize a vulnerable plaque and facilitate its disruption and thrombosis, causing AMI onset.

Although the results are not unequivocal, due to methodological differences, a body of evidence suggests the activation of the inflammatory pathways and increase in plasma concentrations of inflammatory biomarkers, such as C-reactive protein, and proinflammatory cytokines interleukin-6 and tumor necrosis factor-α, as a result of acute sleep deprivation [27,28,29], particularly in the presence of chronic sleep disturbance [27]. Acute partial sleep deprivation has also been associated with a rise in sympathetic and decrease in parasympathetic cardiac autonomic modulation [28,30,31], and increased circulatory levels of norepinephrine and epinephrine [32], increased heart rate [29,33], and increased blood pressure [33,34]. An increased cardiac contractility, expressed through peak systolic circumferential and longitudinal strain, has been described after 24-hr on-call duty and work-related short-term partial sleep deprivation in young and healthy medical professionals [33].

A single night of sleep restriction of <4 h compared to >7 h of sleep was associated with a significantly reduced coronary flow reserve, an indicator of the coronary microcirculation status [35]. Sleep restriction of two-thirds the usual sleep length (5 h/night) was associated with impaired flow-mediated (endothelium-dependent) vasodilation [36]. Other studies confirmed impaired endothelial function [37] and reported increased arterial stiffness [34]. These mechanisms favor the development of coronary vasoconstriction, which can contribute to plaque disruption, and more persistent and occlusive coronary atherothrombosis.

Considering the coagulation system, a study in healthy medical staff showed that serum cyclooxygenase-metabolized eicosanoid mediators, particularly thromboxane B2—a stable metabolite of thromboxane A2 and the most important marker of platelet activation—were significantly higher after the night shift than at baseline [38]. Therefore, an increased activation state of three body systems could induce the prothrombotic forces associated with partial sleep deprivation: systemic inflammation, sympathetic activity, and platelet activation factors.

Given the above mechanisms, can DST be characterized as a distinct risk factor for AMI? It seems that DST itself is less a direct risk factor, but rather an amplifying, catalysing factor that paves the way so that other factors, i.e., actual triggers, become more pronounced. By destabilising the internal clock, disturbing the entrainment process, compromising the duration and quality of sleep, and by causing emotional distress, DST enhances internal triggering mechanisms mediated by the activation of the sympathetic nervous system. We may also assume that behavioural changes additionally play a role in the post-transitional cardiovascular risk. For example, some individuals may increase their cigarette or alcohol consumption, particularly late chronotypes who are more prone to such behaviours irrespective of DST [39,40,41]. In contrast, both fixed and slowly varying characteristics and factors, including sex, age, and ethnicity, and chronic risk factors, such as hypertension, diabetes, hypercholesterolaemia, and obesity, or medical history, do not act as actual triggers of acute cardiac events. Instead, these factors make a person susceptible to AMI over a long period of time [1,2]. Accordingly, confounding by such factors can be largely eliminated in terms of acute AMI-triggering in the days after the DST transition. Yet, as these triggering mechanisms cannot currently be completely separated, additional experimental, clinical, and epidemiological research is needed to shed more light on this complex issue.

## 6. Possible Role of Cardiovascular Medication

Several studies suggest a protective effect of calcium-channel and ß-blockers against AMI after the DST transitions [5,6,8], and the possibility of an opposite effect of angiotensin-converting-enzyme inhibitors [6,8]. Calcium-channel blockers effectively reduce short-term within-individual blood pressure variability, whereas angiotensin-converting-enzyme inhibitors increase this variability, particularly at higher doses [42]. General suppression of sympathetic activity and prevention of exercise-induced rises in heart rate, blood pressure and rate pressure, produce decreased myocardial contractibility and oxygen consumption, which are the favorable cardiovascular mechanisms of ß-blockers [43]. By influencing the response of the cardiovascular system to psychological, physical, and environmental stimuli, antihypertensive drugs may modify the cardiovascular risk around DST transitions. The observations of the post-transitional risk modification by cardiovascular drugs further support the role of biomechanical and hemodynamic forces as important underlying mechanisms for this phenomenon.

## 7. Future Research

How to best explore this phenomenon in the future? Briefly, we should explore whether the cardiovascular risk associated with the DST transitions is currently underestimated, whether the risk after the spring transition lasts longer than one week only, whether late chronotypes are more affected by DST, and what other individual characteristics may modify the risk.

First, large, prospective, multicenter, multiregional, and long-term studies should include patients admitted because of AMI as well as other types of fatal and nonfatal cardiovascular events, such as cardiac arrhythmias, stress cardiomyopathy, aortic dissection, pulmonary embolism, and stroke. The studies should cover all periods throughout the year, not only the narrow peri-transitional periods in spring and autumn. Prospectively collected data obtained through a predefined questionnaire should include detailed information on chronotype, mental state, and medication used and other individual characteristics, as described below. It would be particularly important to record the exact time and date of an acute disease onset, because in retrospective analysis of data from large registries the recorded time is based on the moment of hospitalization or on the moment of establishment of a person’s death, which often does not correspond to the actual time-point of the event onset [5].

Second, in both prospective and retrospective studies, the control periods should not include the four weeks after the spring DST. However, two, three, or four post-transitional weeks in the spring should be investigated as potential hazard periods, especially for late chronotypes. Instead, control periods should include the remaining days and weeks of the year. For statistical analyses, the designs that may be deployed include a cross-based matrix, which combines estimates of associations defined by multiple parameters and is applied for either non-linear or lagged dependencies [44], generalized additive Quasi–Poisson models to accommodate a Poisson distribution with overdispersion for the daily cases [45], and interrupted time series regression analysis with a function modelling for the seasonality [46]. At the same time, these designs would compensate for the seasonal variation in AMI incidence, since this variation has been well-documented in all climatic regions, and generally is highest in winter and lowest in spring [47,48].

Third, the risk of acute cardiovascular events around the DST transitions should be stratified by chronotype. Chronotype calculations commonly stem from subjective sleep time measures [11,49] or self-ratings [50], but not from objective circadian phase assessments (from, e.g., melatonin). Subjective approaches are well suited for phenomenological studies to, for example, explore parameter differences at the group level (average late compared to average early types). But, given the imprecision in how well humans report their sleep timing, subjective methods are less suitable to design personalized interventions for prospective studies [13]. The current gold standard to assess the circadian phase is the dim light melatonin onset (DLMO) [51,52]. To calculate DLMO from melatonin levels, a series of saliva (or blood) samples must be collected explicitly in the evening hours in dim light conditions [53]. Such protocols are laborious and sometimes impossible to adhere to in field studies and furthermore, are inconvenient for translations into the practice. A recently published novel approach, involving markers derived from blood monocytes (and which since has been developed into a simple hair follicle test), represents an objective assessment of an individual’s chronotype, that is equivalent to the DLMO but only requires a single assessment instead of a series of samples, irrespective of the time of day and irrespective of the lighting environment [26,54]. Related protocols are much less laborious and much easier to adhere to in field studies and, in particular, are very convenient for translations into the practice. Such a more reliable chronotype stratification may help answer whether chronotypes at one end of the distribution might be more disadvantaged, specifically late chronotypes in spring, and chronotypes at the other end of the distribution might be less disadvantaged or even profit from the DST transition. Whether this possibility is additionally modified by different geographical locations also requires deeper exploration.

Fourth, one must thoroughly investigate whether factors like shift work and employment status, region-specific cultural norms, personality factors, cardiovascular medication, psychiatric disorders, dietary behavior, habitual level of physical activity, and other lifestyle factors serve as potential risk mediators and influence the risk in post-DST periods.

Fifth, it should be investigated whether behavioral changes of everyday routines could also modify the post-transitional risk. Relevant studies reported fewer AMI patients with previously verified ventricular arrhythmias [9] and fewer AMIs triggered by physical exertion [5] in the days after the spring transition. In this light, it has been hypothesized that individuals with previous symptoms or known heart disease might modify their usual activities due to DST-associated mood changes, sleepiness, and fatigue, which affects their risk of AMI during the post-transitional period [55].

## 8. Conclusions

The subtle distress caused by circadian misalignment following the DST transition is barely perceived and not easily measured. It arises from the body’s ability to cope with circadian disruption and sleep loss, which appears to adversely affect various biological functions and health by increasing the risk of cardiovascular and immune-related diseases, injuries, mental, and behavioral disorders [56,57]. A body of evidence linking late chronotypes with greater levels of social jetlag, generally greater cardiovascular risk, and longer adaptation to the spring DST transition, implicates that this issue should be more thoroughly investigated. At the moment, the most plausible conclusions for the risk of AMI after the spring DST transition are: (1) the risk is increased, (2) a relatively modest risk increase could be currently underestimated or in some studies undetected due to methodological shortcomings, (3) late chronotypes and/or individuals with high levels of social jetlag could be more affected by the phenomenon, and (4) underlying pathophysiological mechanisms should be further explored.

Many countries worldwide including Argentina, Armenia, Belarus, Brazil, Egypt, Iceland, Iraq, Jamaica, Kazakhstan, Mongolia, Namibia, Russia, Sudan, Turkey, and Uruguay have already abolished DST. In September 2018, the European Commission proposed the same action for the European Union, and in March 2019 the European Parliament voted to abolish DST by 2021. However, we are now aware that 2021 is not the last year ever for moving clocks in the EU and there is no confirmation that the practice will be abandoned soon. The complex bureaucratic mechanisms of implementation of the European Parliament’s decision were additionally complicated by Brexit. If the Republic of Ireland follows the rest of the EU in abandoning DST and British-governed Northern Ireland does not, it would create two different time zones on one island. Probably much more important, the COVID-19 pandemic outbreak and keeping the economy and health systems running have become the outmost priorities for EU countries.

A considerable number of questions and uncertainties relate to the actual impact of DST on human health. Regardless of the development in the EU, given the large number of countries outside the EU that still apply DST, a significant part of the world’s population will continue to be affected by this biannual clock change. Therefore, the question of increased AMI risk in the post-transitional period remains an intriguing public health issue. Improved and more accurate exploration of the possible impact on AMI in populations affected by DST could show that such an adverse impact is more significant than we currently think.

## Figures and Tables

**Table 1:** Results of the individual studies investigating the risk of AMI after the DST transitions.

Study [Reference Number]	Incidence Risk Ratio (95% Confidence Interval) of AMI (Post-Transitional Week vs. Control Weeks)	
	Spring (to DST)	Autumn (from DST)
Janszky and Ljung, 2008 [3]	1.051 (1.032–1.071)	0.985 (0.969–1.002)
Janszky et al., 2012 [6]	1.039 (1.003–1.075)	0.995 (0.965–1.026)
Čulić, 2013 [5]	1.15 (1.04–1.26)	1.19 (1.07–1.32)
Jiddou et al., 2013 [4]	1.17 (1.00–1.36)	0.99 (0.85–1.16)
Kirchberger et al., 2015 [8]	1.077 (0.981–1.182)	1.025 (0.928–1.133)
Sipilä et al., 2015 [9]	1.01 (0.96–1.07)	0.99 (0.94–1.04)
Derks et al., 2021 [7]	0.99 (0.94–1.05)	1.00 (0.95–1.06)

## Data Availability

Not applicable.
